# Arterial Stiffness in Nonhypertensive Type 2 Diabetes Patients in Ghana

**DOI:** 10.1155/2016/6107572

**Published:** 2016-09-28

**Authors:** Kwame Yeboah, Daniel A. Antwi, Ben Gyan

**Affiliations:** ^1^Department of Physiology, School of Allied & Biomedical Sciences, University of Ghana, Accra, Ghana; ^2^Department of Immunology, Noguchi Memorial Institute for Medical Research, University of Ghana, Legon, Ghana

## Abstract

*Background*. Increased arterial stiffness is an independent cardiovascular risk factor in diabetes patients and general population. However, the contribution of diabetes to arterial stiffness is often masked by coexistent obesity and hypertension. In this study, we assessed arterial stiffness in nonhypertensive, nonobese type 2 diabetes (T2DM) patients in Ghana.* Methods*. In case-control design, 166 nonhypertensive, nonobese participants, comprising 96 T2DM patients and 70 nondiabetes controls, were recruited. Peripheral and central blood pressure (BP) indices were measured, and arterial stiffness was assessed as aortic pulse wave velocity (PWVao), augmentation index (AIx), cardioankle vascular index (CAVI), and heart-ankle pulse wave velocity (haPWV).* Results*. With similar peripheral and central BP indices, T2DM patients had higher PWVao (8.3 ± 1 versus 7.8 ± 1.3, *p* = 0.044) and CAVI (7.9 ± 1.2 versus 6.9 ± 0.7, *p* = 0.021) than nondiabetic control. AIx and haPWV were similar between T2DM and nondiabetic controls. Multiple regression models showed that, in the entire study participants, the major determinants of PWVao were diabetes status, age, gender, systolic BP, and previous smoking status (*β* = 0.22, 0.36, 0.48, 0.21, and 0.25, resp.; all *p* < 0.05); the determinants of CAVI were diabetes status, age, BMI, heart rate, HbA1c, total cholesterol, HDL cholesterol, and previous smoking status (*β* = 0.21, 0.38, 0.2, 0.18, 0.24. 0.2, −0.19, and 0.2, resp.; all *p* < 0.05).* Conclusion*. Our findings suggest that nonhypertensive, nonobese T2DM patients have increased arterial stiffness without appreciable increase in peripheral and central pressure indices.

## 1. Introduction 

Type 2 diabetes (T2DM) is a major risk factor for the development of cardiovascular diseases (CVDs) [1]. It is estimated that, in the next 20 years, sub-Saharan Africa will have the highest growth in the number of people with diabetes among all other regions of the world, and this will increase the CVD burden [2, 3]. Traditional risk factors that predict CVD events fail to account for the excess CVD mortality found in T2DM patients. A number of studies have identified abnormalities of arterial function in diabetes patients [4–7], and it is now recognized that aortic stiffness, measured by pulse wave velocity (PWV), is highly predictive of cardiovascular and all-cause mortality in subjects with type 2 diabetes and general population, possibly across all age range [8].

Assessment of arterial stiffness in T2DM patients using pulse wave velocity (PWV) is often confounded by coexistent hypertension and blood pressure variations at the time of measurement. In both diabetes and nondiabetes subjects, PWV is strongly associated with age and blood pressure (BP) [4]. However, in sub-Saharan African population, contrary to Caucasians, BP accounts for higher variation of PWV than chronological age [9]. Cardioankle vascular index (CAVI) is a measure of arterial stiffness, reported to be less dependent on BP [10] and computed using two stiffness indices: *β*-stiffness and Bramwell-Hill's formula [11]. The high prevalence of hypertension in T2DM patients makes the contribution of diabetes* per se* to arterial stiffness difficult to ascertain. In this study, we compared the levels of arterial stiffness in nonhypertensive, nonobese T2DM patients to age- and gender-matched nondiabetes controls. We hypothesize that, compared to age- and gender-match controls, nonhypertensive, nonobese T2DM patients would have higher levels of arterial stiffness.

## 2. Methods

### 2.1. Design and Subjects

The study was conducted at the National Diabetes Management and Research Centre, Korle-Bu Teaching Hospital, Accra, Ghana. A total of 166 nonhypertensive, nonobese volunteers, comprising 96 T2DM patients and age- and gender-matched 86 nondiabetes controls within the ages of 35–75 years, were recruited for the study. T2DM was assessed clinically; the patients were diagnosed with diabetes after 30 years of age and treated initially with lifestyle management or oral hypoglycaemic drugs for at least 2 years. T2DM patients were selected by systematic sampling as every 4th consenting patient visiting the clinic. Based on the gender and age decade of T2DM patients, nondiabetes controls were randomly recruited from the surrounding communities into the study. All the nondiabetes volunteers were screened using fasting plasma glucose (FPG) and 2-hour post-75 g glucose load plasma glucose (2 h PPG) to confirm their nondiabetes status. In all, 95 nondiabetes, nonobese volunteers with no history of hypertension were enrolled into the study. However, 9 participants were excluded from analysis; 6 had impaired fasting glucose and 3 were impaired glucose tolerant (too few to be analysed!). Individuals with foot ulcers, established CVDs, BMI ≥ 30 kg/m^2^, history of hypertension treatment, and/or brachial blood pressure >140/90 mmHg were excluded from the study. Ethical approval for this study was given by the University of Ghana Medical School Ethical and Protocol Review Committee (Protocol ID number MS-Et/M.2, P.4.10/2012-2013).

### 2.2. Anthropometric Measurements

Using standard protocols [12], body weight was determined twice using a homologated electronic scale (Seca 770, Hamburg, Germany) following due calibration (precision ± 0.1 kg), with the patient wearing light clothing and shoes removed. Height was also measured with a portable system (Seca 222, Hamburg, Germany) with the patient shoeless in the upright position. Body mass index (BMI) was calculated as weight (kg) divided by height squared (m^2^). Waist circumference was measured with nonelastic tape measure at the upper border of the iliac crest, parallel to the floor without compressing the skin. Body composition monitor (BF-508, Omron Healthcare, Inc., Vernon Hills, IL, USA) was used to assess percentage body.

### 2.3. Biochemical Analysis

Blood samples were drawn in the morning, after 8–12 hours of overnight fasting, into plain vacuum tubes to measure plasma lipids and fluoride oxalate tubes for glucose levels. FPG, 2 h PPG, total cholesterol (TC), high-density lipoprotein (HDL) cholesterol, and triglyceride (TG) levels were analysed by colorimetric enzymatic assays using BS 120 chemical autoanalyzer (Mindray, China) and commercial reagents (Randox Laboratory Reagents, UK). Low-density lipoprotein cholesterol levels were calculated using Friedewald's formula [13]. HbA1c was assayed using affinity chromatography on PDQ Plus HPLC autoanalyzer (Primus Diagnostics, Trinity Biotech, Ireland). All analyses were performed at the Diabetes Research and Chronic Disease Reference Laboratory.

### 2.4. TensioMed Arteriograph Measurements

Arteriograph (TensioMed Kft., Hungary) was used to measure aortic pressure indices, aortic PWV (PWVao), and aortic augmentation index (AIx), with the subject lying supine for at least 10 minutes in a temperature controlled room (22 ± 2°C). The Arteriograph cuff was applied on the right arm over the brachial artery to detect arterial wall oscillations in the upper arm using the “stop-flow” principle previously described [14]. To determine aortic PWV, the Arteriograph uses the physiological behaviour of the wave reflection; the ejected direct (first systolic) pulse wave is reflected back mostly from the aortic bifurcation. The device measures the time interval between the peaks of the direct (first) and reflected (late) systolic wave (return time). For both the invasive and noninvasive aortic PWV computations, the aortic length is measured as distance from sternal notch (jugulum) to the upper edge of the pubic bone (symphysis). Stiffer arteries are less elastic and transmit pulse waves faster, reflected earlier at the abdominal aortic bifurcation, resulting in decreased pulse return time and, hence, higher PWV [15, 16]. To avoid overestimation of aortic path length by body surface contortions, a specialized body caliper was used to measure jugulum-symphysis distance.

PWVao was calculated by using the formula:

(1) $$\begin{array}{c} \text{PWVao }\left(\;m/s\;\right)=\frac{2\cdot \left(jugulum\text{-symphysis distance}\right)}{\text{Return time}}. \end{array}$$

Augmentation index (AIx) is computed using the following algorithm:

(2) $$\begin{array}{c} AIx=\frac{P_{2}-P_{1}}{PP}\times 100, \end{array}$$

where *P*_1_ is the amplitude of the first (direct) wave, *P*_2_ is the amplitude of the late (reflected) systolic wave, and PP is the pulse pressure.

### 2.5. Vasera Measurements

CAVI and heart-ankle (ha) PWV were measured using the Vasera 1500 N (Fukuda-Denshi, Japan) with the participant resting supine for at least 10 minutes before the measurement. Electrocardiogram electrodes were placed on both wrists, a microphone for detecting heart sounds was placed on the sternum, and cuffs were wrapped around the upper arms and the ankles. CAVI values were computed automatically. Briefly, CAVI corresponds to the stiffness parameter *β*, calculated from values of haPWV and BP as follows:

(3) $$\begin{array}{c} \beta =\left(\;\frac{2\rho }{\Delta P}\;\right)\cdot \left[\;\ln\left(\;\frac{P_{s}}{P_{d}}\;\right)\;\right]\cdot {PWV}^{2}, \end{array}$$

where *ρ* indicates blood density; Δ*P* pulse pressure; ln natural log; *P*_*s*_ systolic BP; and *P*_*d*_ diastolic BP [11, 17].

CAVI and Arteriograph measurements were performed in random order using a digital algorithm.

### 2.6. Statistical Analysis

Continuous data were analysed with the Shapiro-Wilk test to determine their distribution. Skewed data were logarithmically transformed. The data were presented as mean ± standard deviation and analysed by Student's *t*-test. Categorical data were presented as counts (percentages) analysed by the *χ* ^2^ test. Multiple regression analysis was performed with PWVao and CAVI, as well as haPWV and AIx, as the dependent variable, and the following parameters were forced into the model as covariates: diabetes status, age, gender, duration of diabetes (for T2DM patients group), BMI, height, previous smoking status, alcohol intake, systolic BP, heart rate, HbA1c, total cholesterol, triglycerides, and HDL cholesterol. *p* values < 0.05 were considered statistically significant.

## 3. Results

**Figure 1:**
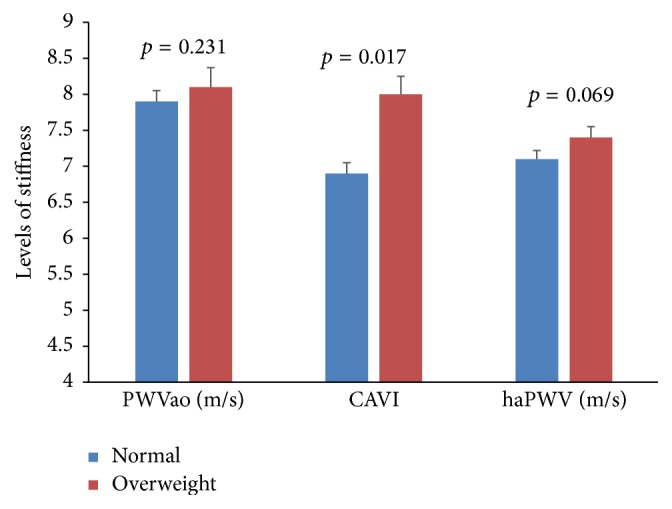
Comparison of arterial stiffness indices between normal and overweight participants.

**Figure 2:**
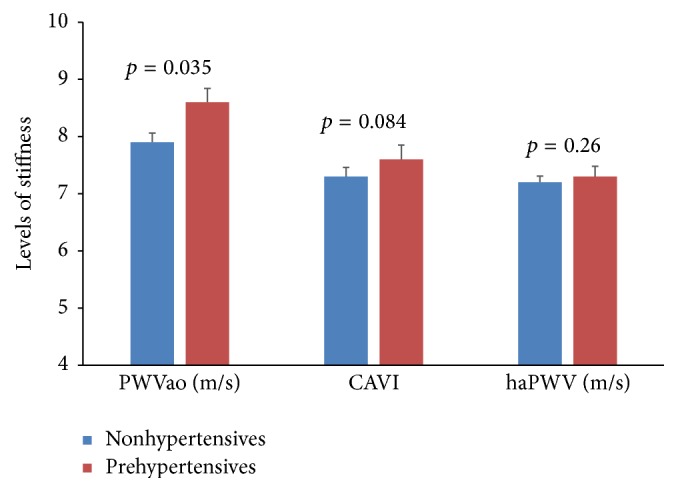
Comparison of arterial stiffness indices between nonhypertensive and prehypertensive participants.

T2DM patients and nondiabetes controls were similar in age, gender distribution, lifestyle (alcohol and previous smoking status), and anthropometric indices. Mean values of AIx and peripheral and central BP indices measured by the Arteriograph were similar between T2DM patients and nondiabetes controls. However, diastolic and pulse BPs measured with Vasera were higher in nondiabetes controls than T2DM patient. Compared to nondiabetes controls, T2DM patients had higher PWVao and CAVI (Table 1). When the study participants were categorised based on BMI, overweight participants (BMI = 25–30 kg/m^2^, *n* = 94) had higher CAVI but similar PWVao and haPWV, when compared to normal participants (BMI ≤ 24.9, *n* = 72; Figure 1). Also, based on BP, prehypertensive (BP = 130–139 mmHg, *n* = 78) participants had higher PWVao than nonhypertensive participants (BP < 130 mmHg, *n* = 88; Figure 2).

In correlational analysis, PWVao increases with increasing age (*r* = 0.33; *p* = 0.005), body fat (*r* = 0.26; *p* = 0.028), brachial systolic pressure (*r* = 0.25; *p* = 0.034), and heart rate (*r* = 0.28; *p* = 0.018). No association was found between PWVao and duration of diabetes (*r* = 0.23; *p* = 0.055), BMI (*r* = 0.13; *p* = 0.272), and waist circumference (*r* = 0.21; *p* = 0.086). CAVI was found to be positively associated with age (*r* = 0.64; *p* < 0.001) and duration of diabetes (*r* = 0.24; *p* = 0.041) but negatively associated with BMI (*r* = −0.26, *p* = 0.025). CAVI did not show any significant association with the components of brachial blood pressure parameters.

In the multiple regression analysis, determinants of PWVao in the entire study population were diabetes status, age, gender, systolic BP, and previous smoking status; in T2DM patients, they were age, duration of diabetes, gender, systolic BP, alcohol intake, and previous smoking status; in nondiabetes controls, they were age and gender. The determinants of CAVI in entire study population were diabetes status, age, BMI, heart rate, HbA1c, total cholesterol, and HDL cholesterol; in T2DM patients, they were age, duration of diabetes, and total cholesterol; in nondiabetes controls, they were age, WHR, systolic BP, heart rate, HDL cholesterol, and previous smoking status (Table 2). Significant predictors of haPWV in the entire study population were age, gender, BMI, WHR, heart rate, HbA1c, total cholesterol, HDL cholesterol, and alcohol intake; in T2DM patients, they were age, duration of diabetes, gender, WHR, systolic BP, heart rate, HbA1c, and alcohol intake; in nondiabetes controls, they were age, HbA1c, total cholesterol, HDL cholesterol, and alcohol intake. Significant determinants for haPWV in the entire study population were age, gender, BMI, WHR, heart rate, HbA1c, total cholesterol, HDL cholesterol, and alcohol intake; in T2DM patients, they were age, duration of diabetes, gender, WHR, systolic BP, heart rate, HbA1c, and alcohol intake; and in nondiabetes controls, they were age, HbA1c, total cholesterol, HDL cholesterol, and alcohol intake. Significant predictors of AIx in the entire study population were diabetes status, age, gender, body height, systolic BP, heart rate, and HbA1c; in the T2DM patients, they were body height, systolic BP, heart rate, HbA1c, and HDL cholesterol; and in nondiabetes controls, they were age, WHR, body height, HDL cholesterol, and alcohol intake (Table 3).

## 4. Discussion

This study provides data on arterial stiffness in nonhypertensive, nonobese indigenous black Africans with T2DM. In this gender and age matched cohort of comparable anthropometric, brachial, and aortic BP indices, we observed increased PWVao and CAVI in T2DM patients compared to nondiabetes controls. The differences in PWVao and CAVI between T2DM patients and nondiabetes controls persisted after multiple adjustments of various risk factors in regression models. The major finding of this study is that T2DM patients have increased stiffness in the arterial wall in the absence of systemic hypertension.

Diabetes is recognized as important risk factor for early arterial aging [8]. Previous studies have shown that T2DM stiffens the aorta and carotid, brachial, femoral, and lower-leg arteries [5, 6]. Our findings are similar to other studies. Compared to healthy controls, arterial stiffness was reported to be higher in T2DM patients in United Kingdom [4, 18]. In the Hoorn study, T2DM patients had increased arterial stiffness, measured ultrasonically as distensibility and compliance, of both elastic (carotid) and muscular (femoral and brachial) arteries [19]. Tedesco et al. also reported synergistic effect of hypertension and T2DM on arterial stiffness, measured as carotid-femoral PWV [20]. In Brazilian population studies [21], PWV was higher in diabetes patients than nondiabetes control when stratified by hypertension status.

It is well established that increased arterial stiffness is responsible for the earlier wave reflections and changes in pressure contours, leading to the elevation of peripheral and central systolic pressures and pulse pressure [22, 23]. In this study, however, compared to the nondiabetes controls, PWVao and CAVI were elevated in the T2DM subjects without accompanying increase in brachial and aortic BPs. This observation may indicate that, in T2DM, arterial stiffening precedes BP increase. The short mean duration of diabetes may imply that, at early phase of T2DM, aortic stiffness occurs without any appreciable increase in peripheral and central pressure indices. Insulin resistance and hyperglycaemia associated with T2DM may explain the increased arterial wall hypertrophy and fibrosis [24] through generation of advanced glycated end products [25], oxidative radicals [26], and proinflammatory cytokines [27]. These findings might be in agreement with the observation that people with T2DM have a 3- to 6-fold increased risk of CVDs compared to the general population and, also, the failure of traditional cardiovascular risk factors to account for the excess CVD risk in these patients [9, 22]. Large arterial stiffness has been demonstrated to be a cardiovascular risk factor in diabetes patients and people impaired glucose tolerance [4, 28]. The major determinants of PWV and CAVI in our study population are similar to what has been published in other studies [4, 5, 29].

In our study, mean AIx was similar among T2DM patients and nondiabetes controls; however, AIx was related to diabetes status after multiple adjustments of several risk factors in the regression model. Other studies have reported that AIx, which was similar between diabetes and nondiabetes subjects, increased in diabetes patients after adjustment of heart rate [29–32]. However, in one study, which included patients with T1DM and T2DM, no changes in AIx between patients and controls were observed after adjustment of heart rate [5]. AIx measures the arterial function, including wall stiffness, the number and location of reflection sites, and the amplitude and timing of the reflected wave. The inverse relationship between body height and AIx supports the previous findings that people with short stature have reduced distance to arterial pressure reflecting sites and this influences the timing and magnitude of arterial wave travel, causing early return to the heart (during systole) and resulting in an increase in AIx [30]. Short stature is independently associated with CVD mortality in longitudinal studies [33, 34] and meta-analysis [35].

In this study, novel equipment, the Arteriograph, was used to measure aortic stiffness. The principle of operation of the Arteriograph had been thoroughly investigated with invasive and noninvasive methods and has been found to be the measure of the stiffness of the central aorta in diabetes patients [36] and nondiabetes subjects [37]. The simplicity of the Arteriograph, its operator-independence and measurement using a single brachial cuff, makes it conducive for normal clinical care in low resource region like sub-Saharan Africa. However, the findings of this study indicate that PWVao measured by Arteriograph can covary with BP making aortic stiffness susceptible to white collar effect. The Vasera, which measures arterial stiffness using a novel stiffness index, CAVI, was found to be less dependent on blood pressure in this study. CAVI has been shown to be a useful tool to screen persons with moderate to advanced levels of arteriosclerosis. Horinaka et al. [38] reported that CAVI was strongly associated with plaque burden measure by coronary intravascular ultrasound in the left main coronary artery of patients with coronary artery disease. Mineoka et al. [39] also showed that CAVI correlated with coronary artery calcification, implying that CAVI could be an evaluation index for macrovascular complication of diabetes. In this study, CAVI was found to be associated with most of the traditional cardiovascular risk factors, raising the possibility that CAVI might predict cardiovascular outcomes in population of African ancestry. However, further longitudinal studies are required to investigate this claim.

The haPWV, from which CAVI was derived, measures the stiffness in central (descending aorta) and muscular arteries. The findings of this study show that haPWV was associated with most CVD risk factor. Diabetes affects the stiffness of central elastic arteries more drastically than peripheral muscular arteries [6], making measurement of peripheral arterial stiffness alone less informative in diabetes patients. Unlike stiffness in central elastic arteries (heart-femoral PWV), peripheral stiffness, measured as femoral-ankle PWV, was associated with neither ischaemic heart disease [40] nor chronic kidney disease [41] in T2DM patients. haPWV is rarely reported in literature, but its analogous stiffness index, the brachial-ankle PWV (baPWV), has been widely studied. baPWV is associated with CVD risk factors [42], and, in one study, baPWV was found to be better associated with left ventricular mass, diastolic functions, and other indices of arterial functions better than carotid-femoral PWV [43]. This may imply that peripheral arterial stiffness adds more information of central elastic arterial stiffness in predicting arterial function and target-organ damage.

## 5. Limitations of the Study

The study was a case-control design with subjects recruited from a specialized clinical facility and nondiabetes controls were conveniently sampled from the general population. Hence, we cannot infer causality from the data and the conclusion cannot be generalized to the entire Ghanaian population. In addition, humoural biomarkers that underline the pathophysiological mechanisms of arterial stiffness were not assayed. Of particular importance is hypogonadism, assessed as low plasma testosterone levels, which has been associated with cardiovascular risk factors such as obesity, insulin resistance, metabolic syndrome, and high BP; all these conditions are associated with increased arterial stiffness [44]. Testosterone has also been shown to decrease the intrinsic tone of vascular smooth muscle in chemically induced diabetes animal models by downregulating the activity of the RhoA/Rho kinase involved in calcium signalling in vascular smooth muscle cells [45]. This may partially explain the observation in male diabetes patients, in which PWV was inversely associated with plasma testosterone and dehydroepiandrosterone sulphate levels. Indeed, a recent study involving adult men without any CVDs reported that low testosterone was associated with aortic stiffness [46].

## 6. Conclusion

In summary, this study has shown that arterial stiffness, measured as PWVao and CAVI, is elevated in T2DM patients in the absence of obesity and hypertension. This increase in arterial stiffness was not accompanied with significant elevation of central haemodynamic pressure indices. Hence, in nonhypertensive T2DM patients, medication reducing arterial stiffness such as angiotensin conversion enzyme inhibitors, angiotensin receptor inhibitors, and calcium channel blockers may be beneficial [47]. Also, possibility of reducing arterial stiffness by screening and treating hypogonadism in T2DM patients may be investigated in future studies [48].

## Figures and Tables

**Table 1:** Clinical characteristics of study subjects.

	T2DM patients (*n* = 96)	Controls (*n* = 70)	*p*
Age	51.5 ± 11.7	50.6 ± 11.1	0.733
Gender (males/females)	40/56	30/40	0.234
Alcohol intake (%)	18.2	22.1	0.078
Previous smokers (%)	14.3	7.8	0.428
Duration of diabetes (years)	2.6 ± 1.8	—	—
*FPG (mmol/L)*	*8.4 ± 3*	*5 ± 1*	*<0.001*
2 h PPG (mmol/L)	—	6.2 ± 2.2	—
HbA1c (%)	**8.3 ± 1.4**	**5.2 ± 0.9**	<0.001
*Total cholesterol (mmol/L)*	*4.1 ± 4.2*	*5.3 ± 1.4*	*<0.001*
Fasting triglycerides (mmol/L)	1 ± 0.5	1.1 ± 0.5	0.310
HDL (mmol/L)	0.8 ± 0.7	0.8 ± 0.5	0.793
*LDL (mmol/L)*	*4.1 ± 1.3*	*2.8 ± 1.3*	*<0.001*
BMI (kg/m^2^)	26.5 ± 4.4	28.5 ± 5.3	0.085
Body fat (%)	30.4 ± 10.1	33.3 ± 13.7	0.305
Visceral fat (%)	11.1 ± 5.1	9.9 ± 3.1	0.165
Waist circumference (cm)	93.9 ± 10.3	93.5 ± 17.5	0.897
Waist-hip ratio	0.91 ± 0.07	0.92 ± 0.19	0.797
Arteriograph measurements			
*PWVao (m/s)*	*8.3 ± 1*	*7.8 ± 1.3*	*0.004*
Aortic systolic BP (mmHg)	116 ± 12	119 ± 11	0.195
Aortic pulse BP (mmHg)	39 ± 13	43 ± 14	0.201
Augmentation index (%)	20 ± 12	25 ± 13	0.138
Systolic BP (mmHg)	124 ± 9	124 ± 10	0.748
Diastolic BP (mmHg)	74 ± 7	74 ± 9	0.977
Pulse BP (mmHg)	50 ± 7	50 ± 8	0.640
Mean BP (mmHg)	116 ± 10	115 ± 11	0.939
*Heart rate (bpm)*	*74 ± 10*	*65 ± 11*	*0.001*
Vasera measurements			
Systolic BP	130 ± 11	133 ± 32	0.613
*Diastolic BP*	*82 ± 8*	*88 ± 12*	*0.039*
*Pulse BP*	*48 ± 9*	*50 ± 12*	*0.04*
Mean BP	98 ± 8	104 ± 14	0.402
*Heart rate*	*73 ± 10*	*65 ± 12*	*0.001*
*CAVI*	*7.9 ± 1.2*	*6.9 ± 0.7*	*0.021*
haPWV (m/s)	7.4 ± 0.8	7 ± 0.8	0.263

BMI: body mass index; BP: blood pressure; HR: heart rate; PWVao: aortic pulse wave velocity; AIx: augmentation index; CAVI: cardioankle vascular index; haPWV: heart-ankle PWV.

**Table 2:** Multiple regression analysis between patients' characteristics versus PWVao and CAVI.

	PWVao		CAVI	
	*β*	*p*	*β*	*p*
All subjects				
Diabetes status	**0.22**	**0.02**	**0.21**	**0.029**
Age	**0.36**	**<0.001**	**0.38**	**<0.001**
Gender	**0.48**	**<0.001**	0.04	0.692
log BMI	0.13	0.185	**0.20**	**0.033**
log WHR	0.06	0.538	0.17	0.067
log height	0.16	0.092	0.05	0.577
log systolic BP	**0.21**	**0.005**	−0.08	0.309
log heart rate	0.05	0.549	**0.18**	**0.026**
Previous smoking	**0.25**	**0.001**	**0.20**	**0.008**
Alcohol intake	0.01	0.894	0.02	0.759
HbA1c	0.01	0.922	**0.24**	**0.007**
log total cholesterol	−0.04	0.659	**0.20**	**0.014**
log triglycerides	0.01	0.985	0.03	0.76
log HDL	0.01	0.974	**−0.19**	**0.014**
T2DM patients				
Age	**0.25**	**0.024**	**2.82**	**0.006**
Diabetes duration	**0.27**	**0.01**	**4.29**	**<0.001**
Gender	**0.37**	**0.006**	−1.46	0.148
log BMI	0.16	0.216	0.31	0.759
log WHR	0.06	0.62	1.16	0.251
log height	−0.01	0.947	−0.84	0.404
log systolic BP	**0.34**	**0.003**	1.37	0.176
log heart rate	0.04	0.7	1.52	0.134
Previous smoking	**0.23**	**0.027**	0.66	0.512
Alcohol intake	**0.20**	**0.043**	1.44	0.155
HbA1c	−0.10	0.424	1.92	0.058
log total cholesterol	−0.01	0.906	**3.15**	**0.002**
log triglycerides	−0.02	0.888	−0.74	0.464
log HDL	0.01	0.902	1.48	0.144
Controls				
Age	**0.42**	**0.012**	**2.98**	**0.005**
Gender	**0.74**	**0.006**	0.12	0.906
log BMI	0.37	0.099	1.70	0.098
log WHR	0.16	0.423	**2.50**	**0.017**
log height	0.34	0.074	0.92	0.363
log systolic BP	0.03	0.886	**2.60**	**0.013**
log heart rate	0.28	0.217	**2.15**	**0.038**
Previous smoking	0.19	0.21	**4.10**	**<0.001**
Alcohol intake	−0.16	0.295	0.57	0.575
HbA1c	0.30	0.109	1.18	0.244
log total cholesterol	0.08	0.625	1.30	0.201
log triglycerides	−0.11	0.591	1.05	0.302
log HDL	−0.08	0.59	**−1.43**	**0.001**

**Table 3:** Multiple regression analysis between patients' characteristics versus haPWV and augmentation index.

	haPWV		AIx	
	*β*	*p*	*β*	*p*
All subjects				
Diabetes status	−0.11	0.246	**0.21**	**0.037**
Age	**0.42**	**<0.001**	**0.14**	**0.018**
Gender	**−0.22**	**0.03**	**0.20**	**0.017**
log BMI	**0.29**	**0.003**	0.01	0.94
log WHR	**0.19**	**0.044**	−0.01	0.911
log height	−0.09	0.315	**−0.44**	**<0.001**
log systolic BP	0.09	0.217	**0.14**	**0.018**
log heart rate	**0.20**	**0.014**	**−0.51**	**<0.001**
Previous smoking	0.14	0.059	−0.03	0.581
Alcohol intake	**0.27**	**<0.001**	0.04	0.439
HbA1c	**0.26**	**0.004**	**0.16**	**0.026**
log total cholesterol	**0.17**	**0.039**	0.07	0.282
log triglycerides	0.16	0.057	0.04	0.504
log HDL	**−0.28**	**<0.001**	0.04	0.469
T2DM patients				
Age	**0.26**	**0.002**	0.03	0.717
Diabetes duration	**0.45**	**<0.001**	−0.02	0.787
Gender	**−0.25**	**0.02**	0.12	0.252
BMI	0.12	0.274	0.05	0.609
WHR	**0.28**	**0.004**	−0.01	0.909
Height	−0.16	0.092	**−0.5**	**<0.001**
Systolic BP	**0.41**	**<0.001**	**0.2**	**0.031**
Heart rate	**0.17**	**0.037**	**−0.61**	**<0.001**
Previous smoking	0.11	0.191	−0.05	0.555
Alcohol intake	**0.17**	**0.026**	0.09	0.262
HbA1c	**0.2**	**0.017**	**0.29**	**0.001**
log total cholesterol	0.12	0.153	0.11	0.212
log triglycerides	0.04	0.71	0.08	0.364
log HDL	0.16	0.064	**0.17**	**0.04**
Controls				
Age	**0.29**	**0.007**	**0.46**	**<0.001**
Gender	−0.09	0.603	0.17	0.304
BMI	0.31	0.052	0.10	0.453
WHR	0.24	0.111	**0.27**	**0.037**
Height	0.06	0.623	**−0.58**	**<0.001**
Systolic BP	−0.12	0.231	−0.05	0.678
Heart rate	0.01	0.93	−0.01	0.936
Previous smoking	−0.06	0.622	−0.13	0.158
Alcohol intake	**0.36**	**0.001**	**−0.19**	**0.044**
HbA1c	**0.30**	**0.009**	0.14	0.242
log total cholesterol	**0.28**	**0.007**	0.18	0.077
log triglycerides	0.01	0.996	−0.02	0.846
log HDL	**−0.35**	**0.003**	**−0.20**	**0.031**

## Abbreviations

PWVao: Aortic pulse wave velocity
CAVI: Cardioankle vascular index
AIx: Aortic augmentation index
haPWV: Heart-ankle pulse wave velocity
BP: Blood pressure
BMI: Body mass index
CVDs: Cardiovascular diseases
T2DM: Type 2 diabetes mellitus
CVDs: Cardiovascular diseases.
